# G Protein‐Coupled Receptor 17 (Gpr17) Enhances Leptin and Insulin Sensitivity in Lean and Obese Mouse Models

**DOI:** 10.1002/oby.70202

**Published:** 2026-05-13

**Authors:** Xun Sun, Connor Mahler, Natalie D. Stull, Ali Nasiri, Baohua Zhou, Varman Samuel, Gerald Shulman, Jonathan N. Flak, Hongxia Ren

**Affiliations:** ^1^ Herman B Wells Center for Pediatric Research, Department of Pediatrics Indiana University School of Medicine Indianapolis Indiana USA; ^2^ Center for Diabetes and Metabolic Diseases, Indiana University School of Medicine Indianapolis Indiana USA; ^3^ Stark Neurosciences Research Institute, Indiana University School of Medicine Indianapolis Indiana USA; ^4^ Indiana Biosciences Research Institute Indianapolis Indiana USA; ^5^ Division of Endocrinology, Department of Medicine Indiana University School of Medicine Indianapolis Indiana USA; ^6^ Yale Diabetes Research Center New Haven Connecticut USA; ^7^ Department of Internal Medicine Yale School of Medicine New Haven Connecticut USA; ^8^ Department of Cellular & Molecular Physiology Yale School of Medicine New Haven Connecticut USA; ^9^ Department of Biochemistry Molecular Biology, and Pharmacology, Indiana University School of Medicine Indianapolis Indiana USA

## Abstract

**Objective:**

Obesity, a major driver for diabetes development, is characterized by insulin and leptin resistance. We previously showed that loss of G protein‐coupled receptor 17 (Gpr17) in specific hypothalamic neurons and the intestine led to better energy balance and glucose metabolism. Our goal is to test whether general loss of Gpr17 enhances insulin and leptin sensitivity.

**Methods:**

We generated germline *Gpr17* knockout mice on both lean (*Gpr17* ^*−/−*^) and obese (*Gpr17* ^*−/−*^; *ob/ob*) backgrounds and characterized their metabolic profile.

**Results:**

*Gpr17* ^*−/−*^ mice exhibited increased energy expenditure and oxygen consumption. Euglycemic‐hyperinsulinemic clamp studies showed enhanced insulin sensitivity in *Gpr17* ^*−/−*^ mice with increased glycogen synthesis and decreased glycolysis. *Gpr17* ^*−/−*^ *ob/ob* mice had increased insulin sensitivity with lower baseline serum insulin and higher response to exogenous leptin treatment with more reduction in feeding and increased pStat3 activation in hypothalamic nuclei.

**Conclusions:**

These findings highlight the inhibitory effect of Gpr17 in leptin and insulin sensitivity, suggesting its potential as a therapeutic target for obesity treatment.

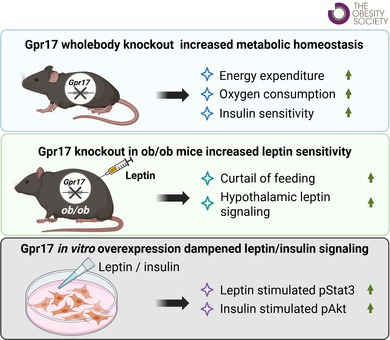

## Graphical abstract

This study identifies G protein‐coupled receptor 17 (Gpr17) as a novel regulator of leptin and insulin sensitivity. Using complementary knockout models, we demonstrate that Gpr17 deletion enhances energy expenditure, restores leptin responsiveness, and improves insulin sensitivity, revealing Gpr17 as a potential therapeutic GPCR target for obesity and related metabolic diseases.

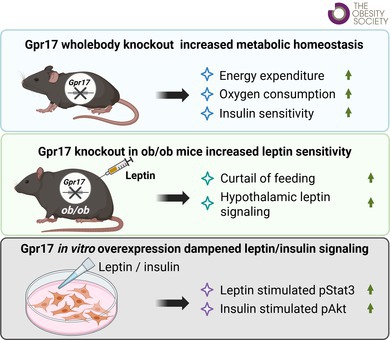

## 1 Introduction

Obesity has risen alarmingly, becoming a major public health challenge worldwide over the past three decades. Obesity is a significant lifestyle‐related condition that poses considerable health risks, including diabetes, fatty liver, cancer, and cardiovascular diseases [1]. There is an urgent need to elucidate the pathophysiology of obesity to develop safe and effective therapeutics for weight loss and maintenance. A long‐term imbalance in energy intake and energy expenditure is the main driver of obesity. The central nervous system (CNS) plays a crucial role in regulating energy balance. Neuronal, endocrine, and metabolic signals converge and integrate into the hypothalamus to regulate homeostatic processes, including feeding and thermoregulation [2]. The most characterized neuronal populations are anorexic proopiomelanocortin (POMC) neurons and orexigenic agouti‐related peptide/neuropeptide‐Y (AgRP/NPY) neurons in the hypothalamic arcuate nucleus (ARC) [3, 4]. POMC neurons produce the neuropeptide POMC, a precursor polypeptide that is processed into several biologically active peptides, including α‐melanocyte‐stimulating hormones (MSH) [5]. α‐MSH activates melanocortin receptors (MC3R and MC4R) on downstream neurons, thereby suppressing appetite and promoting energy expenditure [5]. AgRP neurons exert opposing effects on metabolic regulation by releasing orexigenic peptides NPY and AgRP to promote foraging behavior and food intake [3].

Leptin, a hormone produced and secreted by adipocytes, effectively reduces food intake and body weight by binding to and activating its specific receptor, the long‐form leptin receptor (LepRb) [6]. The activation of LepRb upon leptin binding is a key step in the leptin signaling pathway. It triggers the phosphorylation of Janus kinase 2 (JAK2) and initiates downstream signaling cascades, such as the JAK2/signal transducer and activator of transcription 3 (STAT3) pathway, which mediates most leptin action in the regulation of feeding and energy homeostasis [7]. LepRb is widely expressed in the CNS, particularly in the ARC of the hypothalamus. However, leptin's actions within the CNS are highly interactive and more complex than those limited to ARC, primarily mediated through LepRb‐expressing neurons in multiple brain regions to modulate neuroendocrine outputs [8]. Several hypothalamic regions, including ARC, dorsomedial hypothalamus (DMH), ventromedial hypothalamus (VMH), paraventricular nucleus (PVN), and lateral hypothalamic area (LHA), are either interconnected or engaged in various neural circuits providing different signaling to regulate the overall energy balance [8, 9, 10]. Leptin resistance, a condition that occurs in obesity, is characterized by hyperleptinemia and decreased response to exogenous leptin, limiting the therapeutic efficacy of leptin [11]. Overcoming leptin resistance and increasing leptin sensitization are crucial in clinical obesity treatment.

G protein‐coupled receptors (GPCRs) are the largest family of membrane proteins and represent promising drug targets. GPCR activation plays a critical role in diverse metabolic processes, including glucose homeostasis, insulin sensitivity, hormone secretion, and energy balance [12, 13]. Several GPCRs, such as glucagon‐like peptide‐1 receptor (GLP‐1R), have been identified as targets for obesity treatment and exhibit physiological interactions with leptin signaling [14]. Our previous research demonstrated that G protein‐coupled receptor 17 (Gpr17) functions as an effector of forkhead box class O1 (FoxO1) orexigenic signals in the brain, regulating food intake and peripheral metabolism. *Gpr17* deletion in AgRP neurons and POMC neurons promoted metabolic homeostasis [15, 16, 17]. Moreover, intestinal *Gpr17* knockout contributes to glucose metabolism by modulating GLP‐1 secretion [18, 19, 20]. Based on these findings, we hypothesize that Gpr17 could be targeted for obesity therapy. As our prior studies investigated the physiological functions of Gpr17 only in AgRP neurons, POMC neurons, or the intestine, we examined the overall metabolic consequences of whole‐body Gpr17 deficiency in this study. Although global *Gpr17* knockout mice have been reported previously and shown to display altered metabolic phenotypes [21, 22, 23], the mechanisms by which Gpr17 influences hormonal regulation of energy balance remain poorly defined. Prior studies primarily focused on body weight regulation and food intake during chronic dietary interventions, leaving unresolved whether Gpr17 directly modulates leptin responsiveness and insulin sensitivity, two key pathways that govern systemic metabolic homeostasis. It remains unclear whether Gpr17 signaling influences hypothalamic neuronal responses to leptin or peripheral insulin action in vivo.

In the present study, we addressed this gap using complementary genetic, physiological, and cellular approaches. We examined leptin responsiveness in *Gpr17* knockout mice by assessing leptin‐induced feeding suppression and hypothalamic phosphorylated Stat3 (pStat3) activation across multiple nuclei. We further evaluated systemic insulin sensitivity using hyperinsulinemic‐euglycemic clamp studies and investigated the impact of Gpr17 signaling on leptin and insulin pathways in cultured neuronal cells. In addition, we examined the metabolic response of *Gpr17* knockout mice to an acute high‐fat diet challenge, providing additional insight into how loss of Gpr17 influences energy expenditure and locomotor activity during dietary perturbation. Together, these studies reveal that Gpr17 acts as a negative regulator of leptin and insulin signaling, identifying a previously unrecognized role for this receptor in metabolic regulation. These findings identify Gpr17 as a previously underappreciated regulator of hormonal metabolic signaling and suggest that inhibition of Gpr17 may represent a potential strategy to enhance leptin and insulin sensitivity.

## 2 Methods

### 2.1 Mouse Model

Germline *Gpr17* knockout (*Gpr17* ^*−/−*^) mice were generated as described previously [16]. B6.Cg‐*Lep* ^*ob*^/J (#000632, *ob/ob*) mice were purchased from the Jackson Laboratory (JAX) [24, 25]. *Gpr1* ^*+/−*^ mice were bred with *ob/+* mice to generate *Gpr17* knockout *ob/ob* mice (*Gpr17* ^*−/−*^; *ob/ob*, hereafter KO *ob/ob*) and littermate controls. Normal chow diet (NCD) had 62.1% of calories from carbohydrates, 24.6% from protein, and 13.2% from fat (#5053, LabDiet). High‐fat diet (HFD) contained 60% calories from fat, 20% from protein, and 20% from carbohydrate (#D12492, Research Diets). Experiments were conducted using adult male mice (3–6 months). All animal procedures were approved by the Indiana University School of Medicine Institutional Animal Care and Use Committee.

### 2.2 Metabolic Analyses

Body composition was determined using EchoMRI. Indirect calorimetry measurements were collected using a TSE PhenoMaster Platform as described previously [26]. Briefly, mice were single‐housed and acclimated to metabolic cages for 3 days. After the acclimation phase, data were collected for 3 days under ad libitum feeding conditions, and values from day 3 were presented. Mice were then fasted overnight (16 h) and refed the next morning. Feeding activity was monitored for the next 24 h. To evaluate metabolic responses to a short‐term dietary challenge, *Gpr17* ^*−/−*^ mice and littermate controls were switched from NCD to HFD for 1 week. Mice were subsequently acclimated to metabolic cages for 3 days, followed by continuous recordings for 5 days. Data from day 5 were presented. For the leptin responsiveness experiment, PBS (10 μL/g body weight) was injected i.p. at 7 am and 6 pm for 3 days under ad libitum feeding, and leptin (1 mg/kg) (#AFP1867, National Hormone & Peptide Program) was injected twice daily at the same time points for the next 3 days. Blood samples were collected from tail vein. Serum insulin concentrations were performed using an enzyme‐linked immunosorbent assay (ELISA) kit (#10–1247‐10, Mercodia), while nonesterified fatty acids (NEFA) were determined using NEFA kit (#999–34,691, FUJIFILM).

### 2.3 Hyperinsulinemic‐Euglycemic Clamp

Male wild‐type (WT) and *Gpr17* ^*−/−*^ mice were used for hyperinsulinemic‐euglycemic clamp at the age of 5–7 months after overnight fasting. Further details of the clamp experiment can be found in the following references [27, 28]. Surgery was performed 4–5 days prior to the hyperinsulinemic‐euglycemic clamp to establish a chronic catheter for intravenous infusion of substances (e.g., glucose, insulin) during the clamp. On the day of the clamp experiment, an overnight‐fasted mouse was placed in an oversized restrainer (i.e., rat‐sized) for the experiment to be conducted in an awake and minimally stressed state. A 2‐h hyperinsulinemic‐euglycemic clamp was conducted with a primed‐continuous infusion of human insulin at a rate of 15 pmol/kg/min to raise plasma insulin within a physiological range (~300 pM). Blood samples (20 μL) were collected at 10– to 20‐min intervals for the immediate measurement of plasma glucose concentration, and 20% glucose was infused at variable rates to maintain glucose at basal concentrations (~6 mM). Insulin‐stimulated whole‐body glucose metabolism was assessed with a continuous infusion of [3‐3H]glucose (0.1 mCi/min) throughout the clamp. Basal rates of whole‐body glucose turnover were assessed using a primed‐continuous infusion of [3‐3H]glucose for 2 h prior to the start of the clamp. All infusions were performed using the microdialysis pumps, and all procedures were approved by Yale University Animal Care and Use Committee. To estimate insulin‐stimulated glucose uptake in liver, 2‐deoxy‐D‐[1‐14C]glucose (2‐[14C]DG) was administered as a bolus (10 mCi) at 75 min after the start of the clamp. Blood samples (20 μL) were taken at −5, 80, 85, 90, 100, 110, and 120 min of the clamp for the measurement of plasma [3H]glucose, 3H2O, and/or 2‐[14C]DG concentrations.

### 2.4 qRT‐PCR

Procedure was described previously [29]. Briefly, total RNA was extracted with TRIzol Reagent (#15596026, Invitrogen). Superscript II Reverse Transcriptase (#18064014, Invitrogen) was used to synthesize template cDNA. The specificity of quantitative real‐time PCR (qRT‐PCR) was validated by melting curve analysis on the CFX Connect Real‐Time PCR System (Bio‐Rad). Primer sequences are available upon request.

### 2.5 Immunohistochemistry

Mice were fasted overnight (16 h) and treated with leptin (1 mg/kg, i.p.) (#AFP1867, National Hormone & Peptide Program) 1 h before euthanasia the next morning (9–10 am). Perfusion was conducted with 4% paraformaldehyde (PFA). Brains were processed in 4% PFA overnight and 30% sucrose as described previously [30]. A freezing microtome (Leica) was used to cut brains into 30‐μm sections. Sections were treated sequentially with 1% hydrogen peroxide/0.3% sodium hydroxide for 20 min, 0.3% glycine for 10 min, 0.03% sodium dodecyl sulfate (SDS) for 10 min, and blocking solution (PBS with 2.5% triton, 3% normal donkey serum) for 1 h. The sections were incubated with rabbit anti‐pStat3 or anti‐cFos (#9145, #2250, Cell Signaling Technology) antibodies overnight at room temperature. The next day, sections were incubated with biotinylated donkey anti‐rabbit secondary antibody (#711605152, Jackson ImmunoResearch) followed by avidin HRP (#PK‐6100, Vectastain Elite) and 3,3‐diaminobenzidine (DAB) (#34065, DAB Kit, Thermo Fisher) detection. Images were captured on an Echo Revolve microscope and quantified by ImageJ software.

### 2.6 Cell Culture and Western Blot

Mouse neuroblastoma N2A cells were cultured in Minimum Essential Medium (MEM; #10009CV, Corning) supplemented with 10% fetal bovine serum (FBS; #A3160402, Thermo Fisher) and 1% Antibiotic‐Antimycotic solution (#15240062, Thermo Fisher). Cells were transfected overnight with LepRb together with either CN‐GFP or Gpr17‐GFP plasmids (GeneScript, primer sequences are available upon request) using Lipofectamine 3000 (#L3000015, Thermo Fisher). The following morning, the medium was replaced with MEM containing 10% FBS. After 24 h, cells were switched to serum‐free medium and treated with or without MDL (10 μM) for 20 min. Subsequently, leptin (2 μg/mL) or insulin (10 nM) was added to the respective treatment groups for 30 min or 20 min, respectively. Cells were lysed in total RIPA buffer (1 × PBS, 1% NP‐40, 0.5% sodium deoxycholate, 0.1% SDS), supplemented with Halt Protease and Phosphatase Inhibitor Cocktail (Thermo Fisher), Triton X‐100, and PMSF. Protein concentrations were determined using BCA Protein Assay Kit (Pierce, Thermo Scientific). Equal amounts of protein (20–30 μg) were separated by SDS/PAGE and transferred to PVDF membranes (Immobilon, Millipore). Western blot analyses were performed using antibodies against pStat3, Stat3, pAkt, and Akt (#9145S, #9139S, #4060S, #2920S, Cell Signaling Technology).

### 2.7 Statistical Analyses

Data were analyzed using Student's *t*‐test, one–way ANOVA, or two‐way ANOVA where appropriate with GraphPad Prism software. Group data were presented as mean ± SEM. Statistical significance was defined as *p* < 0.05.

## 3 Results

### 3.1 Germline *Gpr17* Knockout Mice Have Improved Energy Expenditure

**FIGURE 1:**
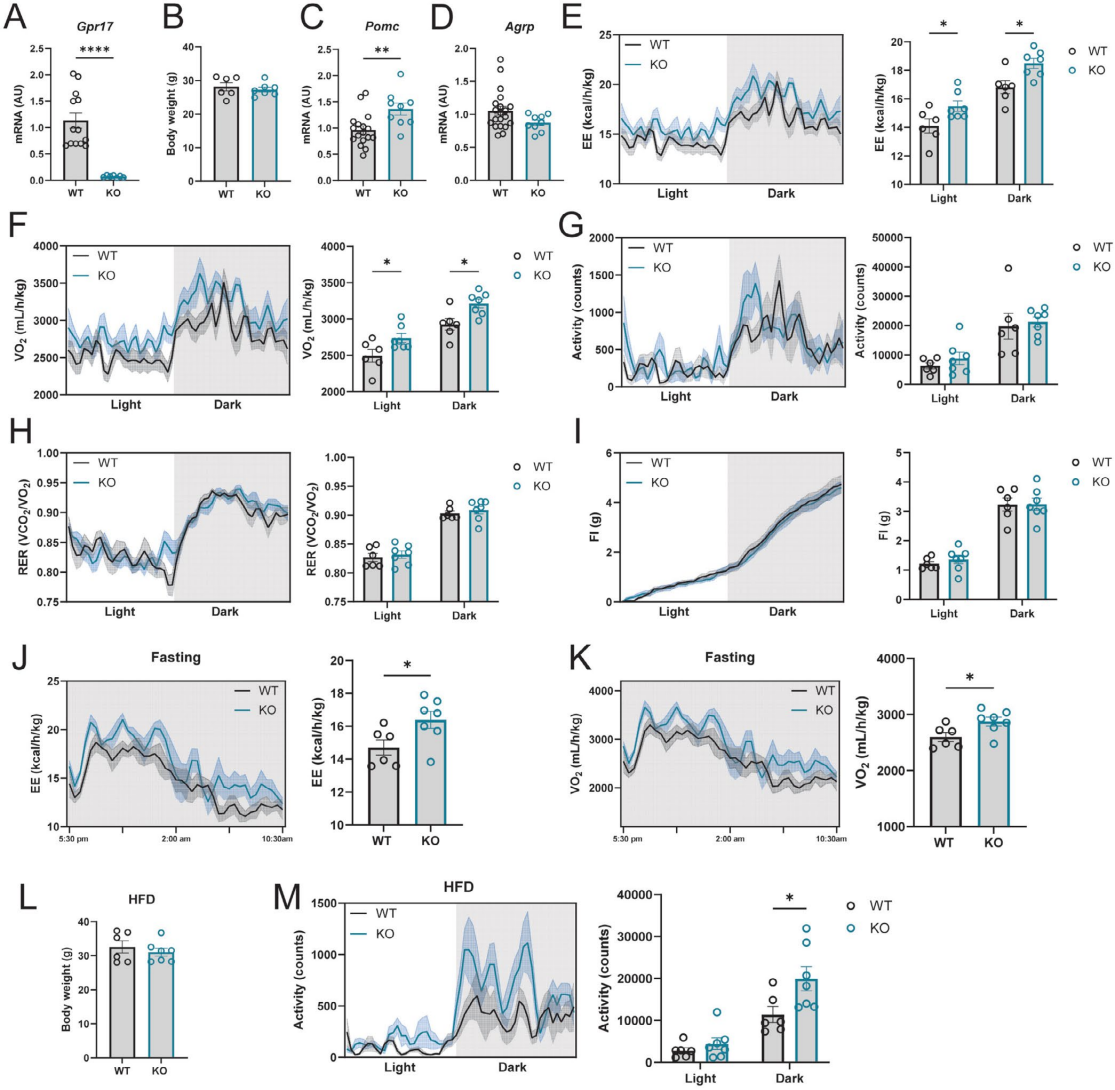
Improved energy expenditure in *Gpr17* germline knockout (KO) mice under NCD. (A) The mRNA levels of *Gpr17* in the hypothalamus (WT = 13, KO = 7). An unpaired two‐tailed Student's *t*‐test was performed. (B) Body weight of adult WT and whole‐body *Gpr17* KO mice (WT = 6, KO = 7). (C, D) The mRNA levels of *Pomc* and *Agrp* in the hypothalamus (WT = 17–20, KO = 9). An unpaired two‐tailed Student's *t*‐test was performed. (E–I) Representative mouse energy expenditure (EE), oxygen consumption (VO_2_), activity, respiratory exchange ratio (RER), and food intake (FI) during ad libitum feeding (WT = 6, KO = 7). Two‐way ANOVA was performed. (J, K) Representative mouse energy expenditure and oxygen consumption recordings during night fasting (WT = 6, KO = 7). An unpaired two‐tailed Student's *t*‐test was performed. (L) Body weight of adult WT and whole‐body *Gpr17* KO mice after 1 week of HFD (WT = 6, KO = 7). (M) The activity of WT and whole‐body *Gpr17* KO mice during short‐term HFD (WT = 6, KO = 7). Two‐way ANOVA was performed. *p < 0.05, **p < 0.01, ****p < 0.0001. Data were displayed as means ± SEM. [Color figure can be viewed at wileyonlinelibrary.com]

To evaluate the metabolic effects of Gpr17, we generated germline *Gpr17* knockout mice (*Gpr17* ^*−/−*^, KO). We first validated successful ablation of *Gpr17* transcript through mRNA expression analysis (Figure 1A). Adult male wild‐type (WT) and KO mice exhibited comparable body weight (Figure 1B). We next profiled the mRNA levels of orexigenic and anorexigenic neuropeptides in hypothalamic samples. KO mice demonstrated significantly elevated mRNA expression of anorexigenic neuropeptide *Pomc*, a critical regulator of appetite suppression and energy expenditure (Figure 1C). Orexigenic *Agrp* levels were lower in KO hypothalamic samples, though not statistically significant (Figure 1D). Metabolic characterization of WT and KO mice with normal chow diet feeding (NCD) was conducted under ad libitum and overnight fasting conditions. KO mice displayed elevated energy expenditure and oxygen consumption (VO_2_) during both light and dark phases under ad libitum feeding (Figure 1E,F). No statistically significant differences were detected in locomotor activity, respiratory exchange ratio, or food intake (Figure 1G–I). Interestingly, the increased energy expenditure and VO_2_ in KO mice persisted during overnight fasting compared to WT mice (Figure 1J,K). After a short‐term (1‐week) transition from NCD to high‐fat diet (HFD), body weight remained comparable between WT and KO mice (Figure 1L). During HFD feeding, KO mice exhibited more locomotor activity compared to WT mice (Figure 1M). Although no statistically significant differences were observed in energy expenditure or oxygen consumption, these measures consistently trended higher in the KO group than in the WT group. The respiratory exchange ratio and food intake were similar between the two groups (Figure S1A–D).

### 3.2 Leptin‐Deficient *Gpr17* Knockout Mice Are More Sensitive to Exogenous Leptin

**FIGURE 2:**
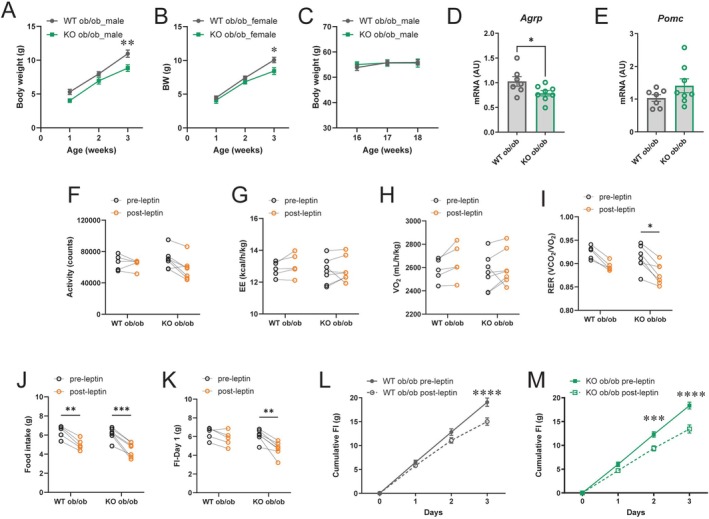
Leptin‐deficient (*ob/ob*) *Gpr17* knockout (KO) mice are more sensitive to leptin‐mediated feeding suppression. (A, B) Growth curves of male (*n* = 11 for each group) and female (WT *ob/ob* = 12–19, KO *ob/ob* = 6) WT and KO *ob/ob* mice from 1 to 3 weeks. Two‐way ANOVA was performed. (C) Growth curves of adult male mice from 16 to 18 weeks (WT *ob/ob* = 6–7, KO *ob/ob* = 8–10). (D, E) *Agrp* and *Pomc* mRNA levels in the hypothalamus (WT *ob/ob* = 7, KO *ob/ob* = 8). An unpaired two‐tailed Student's *t*‐test was performed. (F–J) Individual changes of activity, energy expenditure (EE), oxygen consumption (VO_2_), respiratory exchange ratio (RER), and food intake (FI) in WT and KO *ob/ob* mice after 3 days of leptin treatment (WT *ob/ob* = 5, KO *ob/ob* = 7). Two‐way ANOVA was performed. (K) Individual changes in food intake after the first day of leptin treatment (WT *ob/ob* = 5, KO *ob/ob* = 7). Two‐way ANOVA was performed. (L–M) Cumulative food intake for 3 days before and after leptin treatment (WT *ob/ob* = 5, KO *ob/ob* = 7). Two‐way ANOVA was performed.*p < 0.05, **p < 0.01, ***p < 0.001, ****p < 0.0001. Data were displayed as means ± SEM. [Color figure can be viewed at wileyonlinelibrary.com]

Leptin‐deficient (*ob/ob*) mice are obese and do not produce leptin. We generated *Gpr17* knockout on a leptin‐deficient background (*Gpr17* ^*−/−*^; *ob/ob*, KO *ob/ob*) to assess the metabolic effects of Gpr17 in an obese state. During the neonatal period (1–3 weeks), both male and female KO *ob/ob* mice displayed lower body weight compared to WT *ob/ob* mice (Figure 2A,B). However, the difference in body weight was no longer evident in the adulthood phase (Figure 2C). Hypothalamic mRNA analyses revealed significantly reduced *Agrp* levels in KO *ob/ob* mice, while *Pomc* levels were elevated but not statistically significant (Figure 2D,E). To assess the response of KO *ob/ob* mice to leptin, mice received PBS injections for 3 days followed by a 3‐day leptin treatment. Following leptin administration, WT *ob/ob* mice and KO *ob/ob* mice exhibited comparable locomotor activity, energy expenditure, and oxygen consumption (Figure 2F–H). However, KO *ob/ob* mice displayed a greater reduction in respiratory exchange ratio, indicating a greater reliance on lipid metabolism for energy rather than carbohydrates (Figure 2I). A significant reduction in food intake was recorded in both groups (Figure 2J). Notably, KO *ob/ob* mice, but not WT *ob/ob* mice, showed decreased food consumption from the first day of leptin treatment (Figure 2K). Furthermore, compared to WT *ob/ob* mice, KO *ob/ob* mice exhibited an earlier and more pronounced difference in cumulative food intake (Figure 2L,M). These data showed KO *ob/ob* mice exhibited enhanced sensitivity to leptin‐mediated feeding suppression and increased lipid oxidation.

### 3.3 Gpr17 Activation Inhibits Leptin Signaling

**FIGURE 3:**
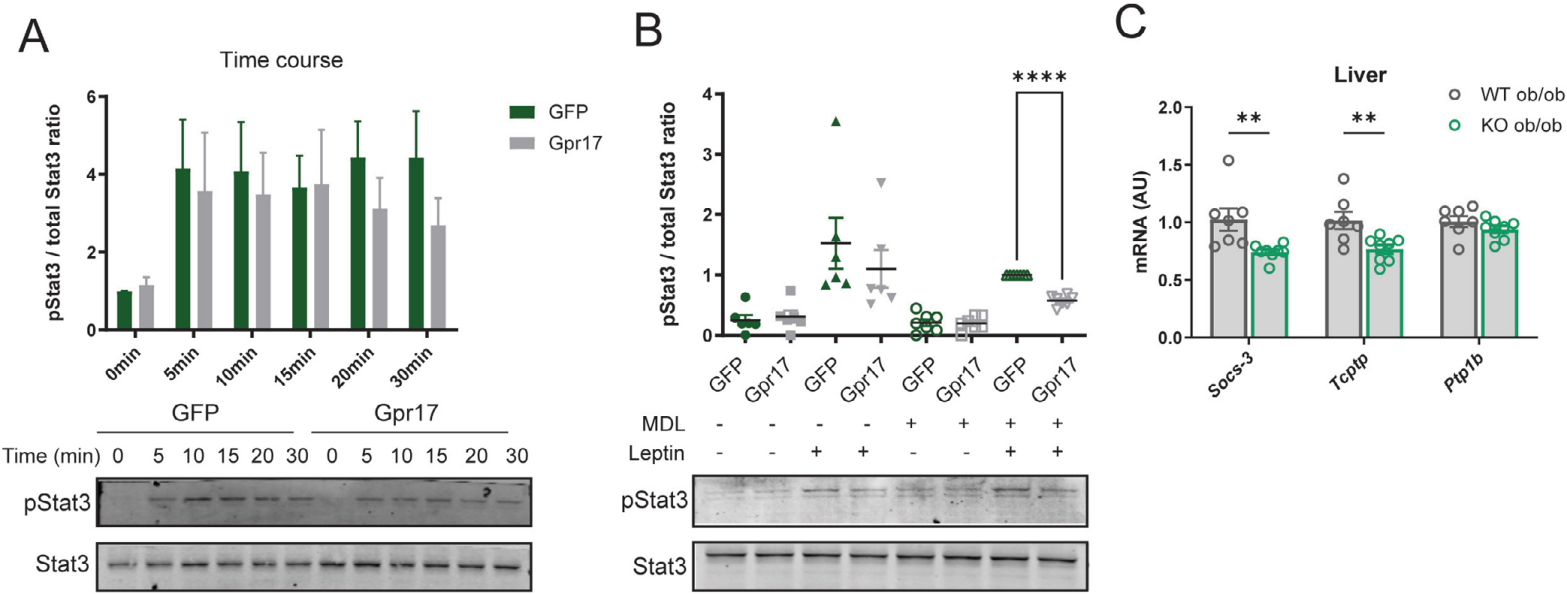
Gpr17 regulates leptin signaling. (A) The time course of detectable pStat3 after leptin treatment in N2A cells (*n* = 4 per each group). (B) Representative Western blotting and quantitative analysis of pStat3 and Stat3 in N2A cells with or without Gpr17 overexpression (*n* = 6–7 per each group). A two‐tailed unpaired *t*‐test was performed. (C) The hepatic mRNA levels of leptin signaling inhibitors in *ob/ob Gpr17* knockout (KO) mice (WT *ob/ob* = 7, KO *ob/ob* = 8). Two‐way ANOVA was performed. **p < 0.01, ****p < 0.0001. Data were displayed as means ± SEM. [Color figure can be viewed at wileyonlinelibrary.com]

To further determine the direct effect of Gpr17 on leptin signaling, we evaluated pStat3 expression in neuroblastoma N2A cells co‐transfected with leptin receptor and control GFP or Gpr17‐GFP plasmids using Western blot analysis. Time‐course data revealed detectable pStat3 levels as early as 5 min post leptin treatment (Figure 3A). After 30 min of leptin treatment, the Gpr17 group treated with the Gpr17 synthetic agonist MDL29,951 showed significantly lower pStat3 levels compared with the GFP control group (Figure 3B), suggesting that Gpr17 activation attenuates leptin signaling. While leptin primarily acts in the hypothalamus to regulate appetite and energy expenditure, it can also affect hepatic metabolism through direct regulation or CNS mechanisms [31]. We evaluated the negative regulators (*Socs3*, *Tcptp*, *Ptp1b*) involved in the leptin signaling pathway in both hypothalamic and hepatic samples from WT *ob/ob* and KO *ob/ob* mice. Although the hypothalamic mRNA levels of these regulators were comparable between genotypes, KO *ob/ob* mice exhibited lower hepatic mRNA expression of *Socs3* and *Tcptp* (Figure S2A and Figure 3C). No significant difference was observed in hepatic gene expression involved in glucose and lipid metabolism (Figure S2B). Thermogenetic gene expression in brown adipose tissue was also comparable between the groups (Figure S2C). These findings suggested that Gpr17 deficiency enhances leptin‐mediated anorexia and energy expenditure through modulation of leptin signaling pathways.

### 3.4 Leptin‐Deficient *Gpr17* Knockout Mice Show Increased Leptin Responsiveness in the Hypothalamus

**FIGURE 4:**
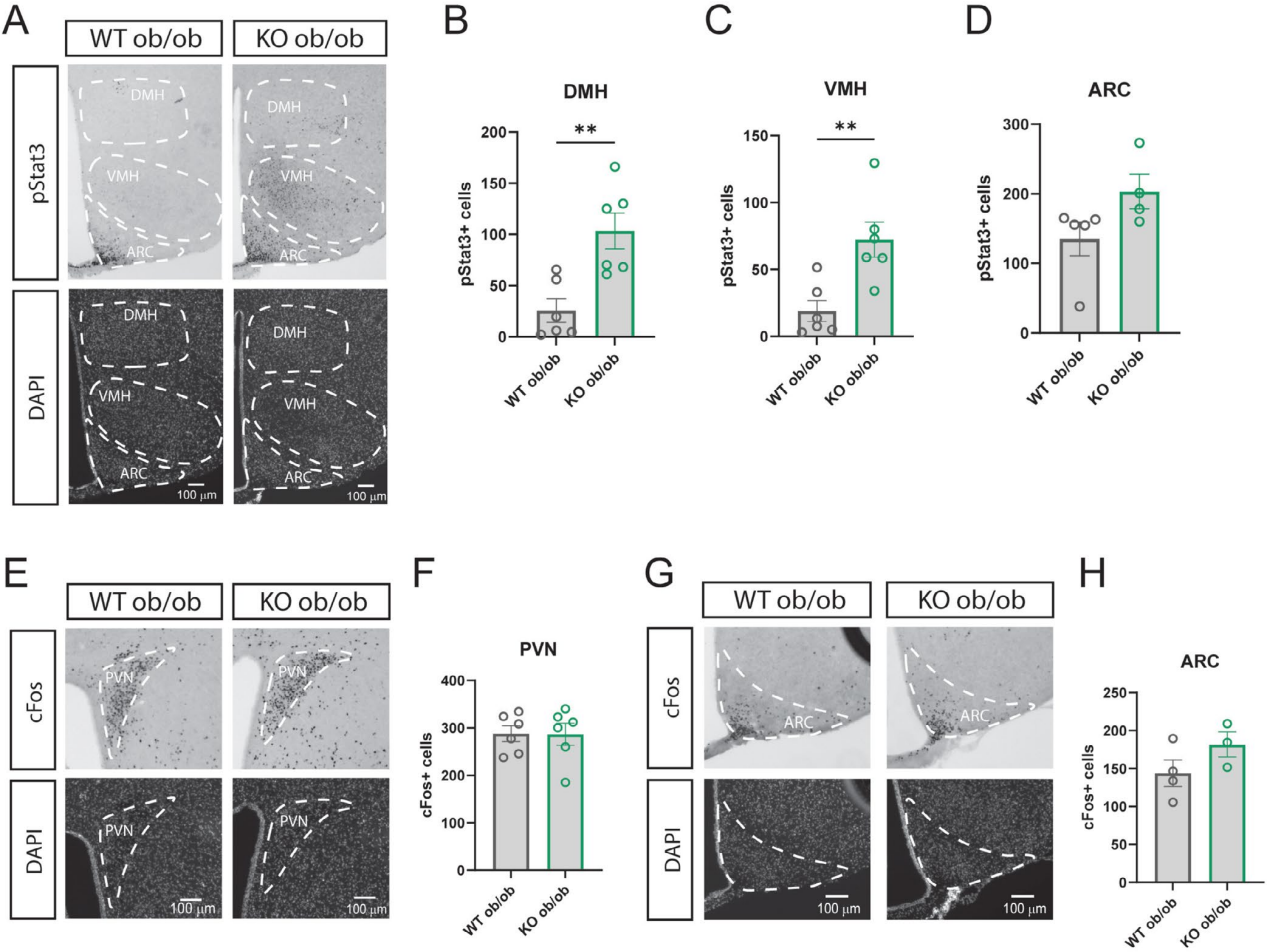
*Gpr17* knockout (KO) o*b/ob* mice show increased hypothalamic leptin sensitivity. (A) Representative images with pStat3 in fasted mice with 1‐h leptin treatment. (B) Quantitative analysis of pStat3‐positive cell number in DMH (*n* = 6 for each group), VMH (*n* = 6 for each group), and ARC (WT *ob/ob* = 5, KO *ob/ob* = 4). A two‐tailed unpaired *t*‐test was performed. (E, F) Representative images and quantitative analysis of cFos‐positive cell number in PVN (*n* = 6 for each group). (G, H) Representative images and quantitative analysis of cFos‐positive cell number in ARC (WT *ob/ob* = 4, KO *ob/ob* = 3). DMH, dorsal hypothalamic nucleus; VMH, ventromedial hypothalamic nucleus; ARC, hypothalamic arcuate nucleus; PVN, hypothalamic paraventricular nucleus. [Color figure can be viewed at wileyonlinelibrary.com]

We next assessed the number of pStat3‐immunoreactive cells in DMH, VMH, and ARC to elucidate leptin signaling activation across these regions (Figure 4A). Fasted KO *ob/ob* mice had significantly increased pStat3‐positive cells in DMH and VMH 1 h after leptin treatment (1 mg/kg) (Figure 4B,C). Although we observed an increased pStat3 immunoreactivity in ARC, this difference did not reach statistical significance (Figure 4D). A representative image of pStat3 immunoreactivity in KO *ob/ob* mice treated with vehicle showed the background staining, confirming that the increased pStat3‐positive cells observed in leptin‐treated KO *ob/ob* mice reflect leptin‐specific signaling (Figure S3). Additionally, we measured cFos immunoreactivity in PVN and ARC as a readout of neuronal activation. The number of cFos‐positive cells in PVN was comparable between the groups (Figure 4E,F), while a higher but not statistically significant level was noted in ARC (Figure 4G,H). These data suggested that KO *ob/ob* mice are more responsive to exogenous leptin in the brain, especially within DMH and VMH.

### 3.5 Gpr17 Deficiency Increases Insulin Sensitivity

**FIGURE 5:**
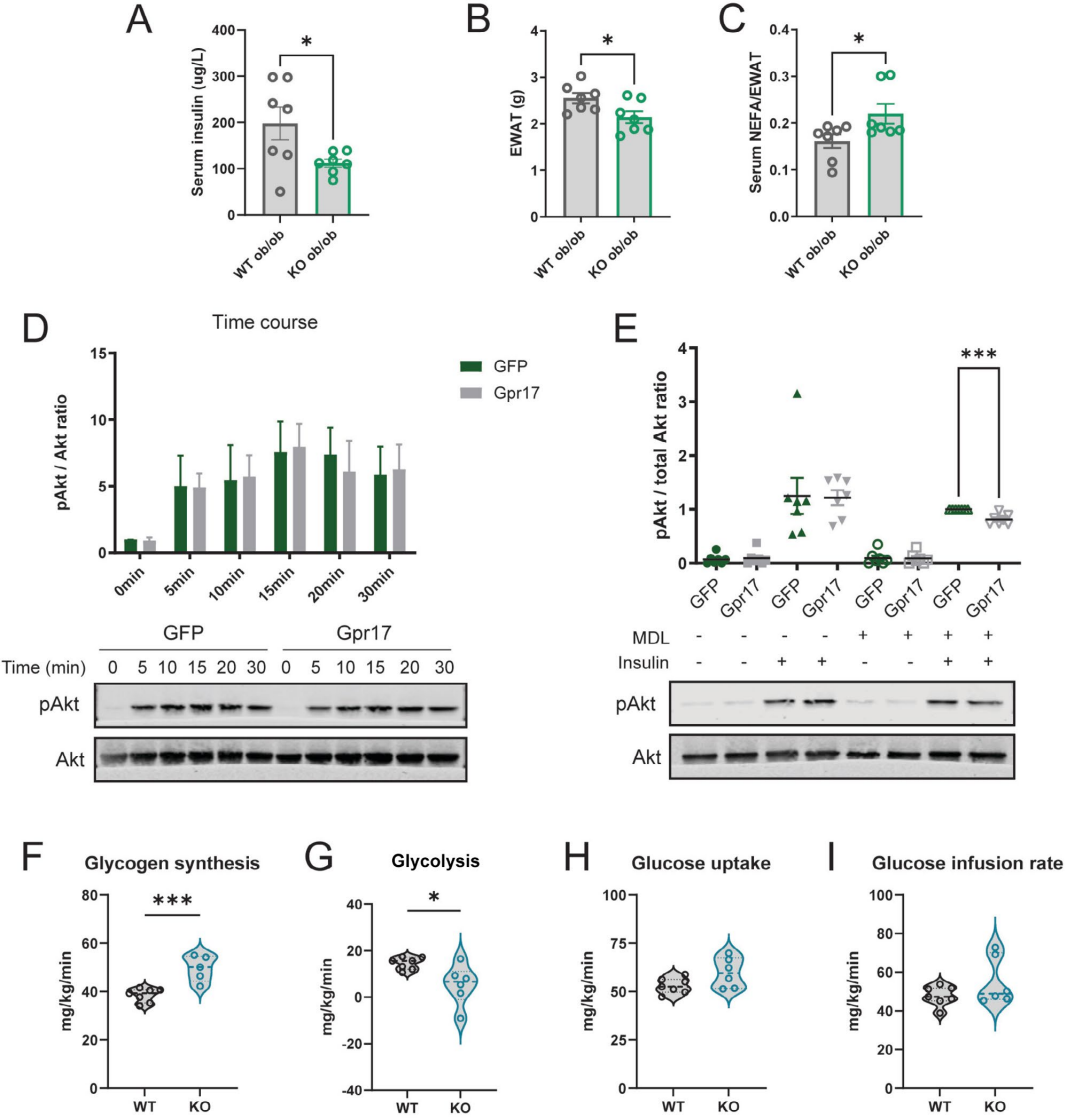
*Gpr17* knockout (KO) sensitizes insulin signaling. (A) Serum insulin levels of WT and KO *ob/ob* mice (*n* = 7 for each group). (B) The weight of epididymal white adipose tissue in WT and KO *ob/ob* mice (*n* = 7 for each group). (C) Normalized serum nonesterified fatty acid (NEFA) levels in WT and KO *ob/ob* mice (*n* = 7 for each group). (D) The time course of detectable pAkt after insulin treatment in N2A cells (*n* = 4 for each group). (E) Representative Western blotting and quantitative analysis of pAkt and Akt in N2A cells with or without Gpr17 overexpression (*n* = 7 for each group). (F–I) Glycogen synthesis, glycolysis, glucose uptake, and glucose infusion rate of whole‐body *Gpr17* KO mice in hyperinsulinemic‐euglycemic clamp (WT = 7, KO = 5–6) An unpaired two‐tailed Student's *t*‐test was performed. *p < 0.05, ***p < 0.001. Data were displayed as means ± SEM. [Color figure can be viewed at wileyonlinelibrary.com]

Insulin resistance, a key feature of obesity and diabetes, is characterized by increased insulin production coupled with a diminished responsiveness to insulin. We assessed the impact of Gpr17 deficiency on insulin sensitivity. KO *ob/ob* mice exhibited lower insulin levels than WT *ob/ob* mice during the ad libitum phase, suggesting improved insulin sensitivity (Figure 5A). Interestingly, although adult KO *ob/ob* mice had comparable body weight with WT *ob/ob* mice (Figure 2B), they had smaller epididymal fat pads (Figure 5B). KO *ob/ob* mice had higher serum nonesterified fatty acids (NEFA) as a percent of epididymal white adipose tissue, suggesting more lipid breakdown (Figure 5C). Next, we evaluated phosphorylated Akt (pAkt) expression in neuroblastoma N2A cells with or without Gpr17 overexpression. Time‐course data revealed that pAkt was detectable after 5 min of insulin treatment (Figure 5D). Addition of MDL29,951 led to a significant decrease in pAkt levels in the Gpr17 group under insulin treatment (Figure 5E). Moreover, *Gpr17* KO mice displayed increased glycogen synthesis and glycolysis in euglycemic‐hyperinsulinemic clamp studies, while glucose uptake and glucose infusion rate remained comparable to WT mice, suggesting increased insulin sensitivity and glucose metabolism (Figure 5F–I).

## 4 Discussion

*Gpr17* deletion in key hypothalamic neuronal populations improves energy balance, while its ablation in enteroendocrine cells (EECs) plays a critical role in glucose metabolism [15, 16, 19]. Given its widespread expression and involvement in metabolic regulation in both the CNS and peripheral tissues, Gpr17 represents a potential target for obesity treatment. In this study, we generated *Gpr17* germline knockout (KO) mice, which displayed enhanced anorexigenic *Pomc* mRNA expression and increased energy expenditure. Leptin‐deficient (*ob/ob*) *Gpr17* KO mice demonstrated enhanced responsiveness to leptin‐induced feeding suppression. The qRT‐PCR analysis revealed reduced expression of the orexigenic peptide *Agrp* in the hypothalamus and lower levels of leptin's negative regulators in the liver of KO *ob/ob* mice compared with WT *ob/ob* mice. Enhanced pStat3 immunoreactivity was observed in the hypothalamus of these mice, particularly in the DMH and VMH. Furthermore, *Gpr17* KO mice showed improved insulin sensitivity. In vitro, Gpr17 activation suppressed both leptin and insulin signaling in cultured cells. Collectively, these findings indicate that *Gpr17* deletion sensitized leptin and insulin signaling and provide potential therapeutic insights for obesity treatment.

Our previous study demonstrated that AgRP neuron‐specific *Gpr17* KO mice exhibit lower body weight accompanied by reduced food intake, increased relative energy expenditure, and enhanced satiety [16]. In contrast, in the present study, global *Gpr17* KO mice showed increased energy expenditure and oxygen consumption but displayed body weight and food intake comparable to WT mice. This divergence suggests that Gpr17 may exert distinct and potentially opposing or compensatory functions across different neuronal populations and peripheral tissues. Alternatively, this could be caused by the different genetic approaches, such as physiological compensation associated with global knockout. The metabolic phenotype of whole‐body *Gpr17* KO mice has been reported by another group [21]. These KO mice showed a trend of less 24‐h feeding on HFD and significantly less feeding activity during the dark phase. Two other groups independently generated *Gpr17* global KO mice and reported leanness, low plasma leptin, and resistance to diet‐induced weight gain [22, 23]. These publications are in line with our findings.

Leptin resistance is characterized by hyperleptinemia and diminished responsiveness to leptin during states of nutritional excess. Restoring leptin sensitivity in obesity has remained a major challenge since leptin's discovery. In our study, *Gpr17* KO mice in the obese state (KO *ob/ob*) showed a greater reduction in food intake following leptin injection, indicating that Gpr17 deficiency enhances leptin responsiveness. Leptin's effect in liver was reported [32]. Hyperleptinemia in obesity was linked to hepatic steatosis by promoting insulin resistance and increasing intracellular fatty acids, thereby driving hepatic inflammation and fibrosis [33]. Whether the reduced hepatic *Socs3* and *Tcptp* expression observed in *Gpr17* KO *ob/ob* mice may alleviate hepatic steatosis through improved leptin signaling remains an important question for future investigation.

It is well established that leptin primarily acts through LepRb‐expressing cells in the brain [34]. Leptin receptors are abundantly expressed in various hypothalamic regions, including the ARC, DMH, and VMH [9, 35]. In addition to well‐characterized LepRb neuron populations defined by transcriptional markers, including POMC, AgRP, and NPY, leptin also acts on γ‐aminobutyric acid (GABA)ergic neurons to influence food intake and energy balance by regulating GABA release through both LepRb‐inhibited and LepRb‐excited GABAergic neuronal subsets, contributing to body weight control [36, 37]. Beyond its direct actions on specific neuronal populations, leptin interacts within complex CNS circuits to modulate metabolic homeostasis. Leptin exerts dynamic effects on neural circuits. Projections from the VMH and DMH to the subparaventricular zone (SPZ) and PVN may mediate leptin's autonomic, endocrine, and behavioral effects [9]. In our study, we observed that leptin induced a significantly higher level of pStat3 in the DMH and VMH of *Gpr17* KO *ob/ob* mice, despite no difference in pStat3 in the ARC and no difference in cFOS in the PVH and ARC. Furthermore, KO *ob/ob* mice exhibited significantly reduced *Agrp* mRNA expression in the hypothalamus. The observed changes in hypothalamic neurons may result from indirect effects. The precise neuronal mechanisms and circuit connectivity through which Gpr17 mediates leptin's effects remain incompletely understood. Future research should aim to clarify the neural circuits involved in these processes.

The GPCR knockout animal studies depend on signaling context and physiological conditions. The usage of pharmacological probes could yield more insight as it was the case for GLP‐1R. The endogenous ligand for Gpr17 remains elusive. For the in vitro cell signaling assays, we used a chemical synthetic compound, MDL29,951, to activate Gpr17 signaling. MDL29,951 is also a potent antagonist for the strychnine‐insensitive glycine binding site of the NMDA receptor (IC50 = 170 nM) [38], which limits its application in vivo. Other groups reported the repurposing of antagonists for cysteinyl leukotriene receptors (CysLTRs) for antagonizing Gpr17 signaling. We recently identified two new chemotypes of Gpr17 antagonists that showed selectivity of Gpr17 over purinergic receptor and CysLTRs [20]. We plan to further develop and optimize these compounds for in vivo applications. However, the work exceeds the current scope of this manuscript and warrants future investigation.

Insulin resistance is a hallmark of diabetes and is associated with obesity [26]. Obesity is further characterized by excessive release of NEFA, glycerol, hormones, and proinflammatory cytokines from adipose tissue. Notably, the release of NEFA is considered one of the most influential factors in modulating insulin sensitivity [39]. Studies have shown that the accumulation of visceral fat correlates with insulin resistance, while subcutaneous fat deposition is associated with higher circulating leptin levels [40]. In our *Gpr17* KO mice, we observed improved insulin sensitivity and enhanced glucose metabolism in euglycemic‐hyperinsulinemic clamp studies. In the obese state, *Gpr17* KO *ob/ob* mice exhibited lower baseline serum insulin levels, which also indicate improved insulin sensitivity. However, the mechanism by which Gpr17 regulates insulin signaling remains unclear. Further investigations are required to elucidate the underlying pathways involved.

There are limitations with our current studies. The global *Gpr17* KO mice display metabolic phenotypes that were not the same as those previously reported in the AgRP neuron‐specific and POMC neuron‐specific mice. Whether there are additional unclear neuronal populations or peripheral tissues that may contribute to the phenotype of whole‐body knockout needs to be further investigated. Second, we used *ob/ob* mice to examine exogenous leptin responsiveness. A potential confounding factor was the *ob/ob* mice's development with leptin deficiency. The absence of leptin during development could cause altered hypothalamic circuits. This animal model represents a rare form of human obesity associated with mutations in the leptin gene. Therefore, a more translationally relevant approach is to use a diet‐induced obesity (DIO) model. Direct assessment of leptin sensitivity in the DIO model is warranted for future studies. Third, we largely used male mice for the study. Whether there is a sexual dimorphism in the whole‐body knockout warrants future investigations.

In summary, Gpr17 ablation enhances both leptin and insulin sensitivity. Targeting Gpr17 through antagonism may offer a novel therapeutic strategy for obesity and its comorbidities.

## Author Contributions

X.S. and H.R. designed and conducted experiments, analyzed data, and wrote and revised the manuscript. C.M. and J.N.F. performed immunohistochemistry. N.D.S. performed TSE experiments. A.N., V.S., and G.S. performed the hyperinsulinemic‐euglycemic clamp experiment. B.Z. contributed to the reviewing and editing. H.R. conceived and supervised the study. All authors reviewed and approved the manuscript.

## Funding

H.R.'s group received funding from the National Institutes of Health (NIH) (R01DK120772, R00DK098294, R03TR003350, and UL1TR002529). The content is solely the responsibility of the authors and does not necessarily represent the official views of the NIH. This publication was made possible by internal awards from the Indiana University School of Medicine. The Yale Mouse Metabolic Phenotyping Center (MMPC) Integrative Physiology Core was supported by Yale MMPC‐Live grant (NIH/NIDDK U2C DK134901).

## Conflicts of Interest

The authors declare no conflicts of interest.

## Supporting information


**Figure S1:** The metabolic features of *Gpr17* germline knockout mice under short‐term HFD (A–D) Representative mouse energy expenditure (EE), oxygen consumption (VO_2_), respiratory exchange ratio (RER), and food intake (FI) during ad libitum HFD feeding (WT = 6, KO = 7).
**Figure S2:** The mRNA expression in *Gpr17* knockout *ob/ob* mice. (A) The hypothalamic mRNA levels of leptin signaling inhibitors in *ob/ob* Gpr17 knockout mice. (B) Hepatic mRNA expression of genes involved in glucose and lipid metabolism. (C) The mRNA levels of thermogenetic genes in brown adipose tissue (BAT). WT *ob/ob* = 7, KO *ob/ob* = 8. Data were displayed as means ± SEM.
**Figure S3:** The representative image of pStat3 immunoreactivity in KO *ob/ob* mice treated with vehicle.

## Data Availability

The data that support the findings of this study are available from the corresponding author upon reasonable request.
