# Supplementary material for: G Protein‐Coupled Receptor 17 (Gpr17) Enhances Leptin and Insulin Sensitivity in Lean and Obese Mouse Models
Source: Obesity (Silver Spring). 2026 May 13;34(Suppl 1):77–87. doi: 10.1002/oby.70202 (PMC13250742; doi:10.1002/oby.70202)
Supplement: Supplementary file 1 — Figure S1: The metabolic features of Gpr17 germline knockout mice under short‐term HFD (A–D) Representative mouse energy expenditure (EE), oxygen consumption (VO2), respiratory exchange ratio (RER), and food intake (FI) during ad libitum HFD feeding (WT = 6, KO = 7). Figure S2: The mRNA expression in Gpr17 knockout ob/ob mice. (A) The hypothalamic mRNA levels of leptin signaling inhibitors in ob/ob Gpr17 knockout mice. (B) Hepatic mRNA expression of genes involved in glucose and lipid metabolism. (C) The mRNA levels of thermogenetic genes in brown adipose tissue (BAT). WT ob/ob = 7, KO ob/ob = 8. Data were displayed as means ± SEM. Figure S3: The representative image of pStat3 immunoreactivity in KO ob/ob mice treated with vehicle. [file OBY-34-77-s001.docx]

SUPPLEMENTARY MATERIALS

**G Protein-Coupled Receptor 17 (Gpr17) Enhances Leptin and Insulin Sensitivity in Lean and Obese Mouse Models**

Xun Sun^1,2,3^, Connor Mahler^4^, Natalie D. Stull^4,5^, Ali Nasiri^6^, Baohua Zhou^1^, Varman Samuel^6^, Gerald Shulman^6,7,8^, Jonathan N. Flak^4,9^, Hongxia Ren^1,2,3^*

^1^Herman B Wells Center for Pediatric Research, Department of Pediatrics, Indiana University School of Medicine; Indianapolis, IN, USA

^2^Center for Diabetes and Metabolic Diseases, Indiana University School of Medicine; Indianapolis, IN, USA

^3^Stark Neurosciences Research Institute, Indiana University School of Medicine; Indianapolis, IN, USA

^4^Indiana Biosciences Research Institute; Indianapolis, IN, USA

^5^Division of Endocrinology, Department of Medicine, Indiana University School of Medicine; Indianapolis, IN, USA.

^6^Yale Diabetes Research Center; New Haven, CT, USA.

^7^Department of Internal Medicine, Yale School of Medicine; New Haven, CT, USA.

^8^Department of Cellular & Molecular Physiology, Yale School of Medicine; New Haven, CT, USA.

^9^Department of Biochemistry, Molecular Biology, and Pharmacology, Indiana University School of Medicine; Indianapolis, IN, USA

*Corresponding author: Hongxia Ren

Email: [renh@iu.edu](mailto:renh@iu.edu)

Phone: 317-274-1567

Postal address: 1210 Waterway Blvd Ste 4100 Indianapolis, IN 46202

**Figure S1**


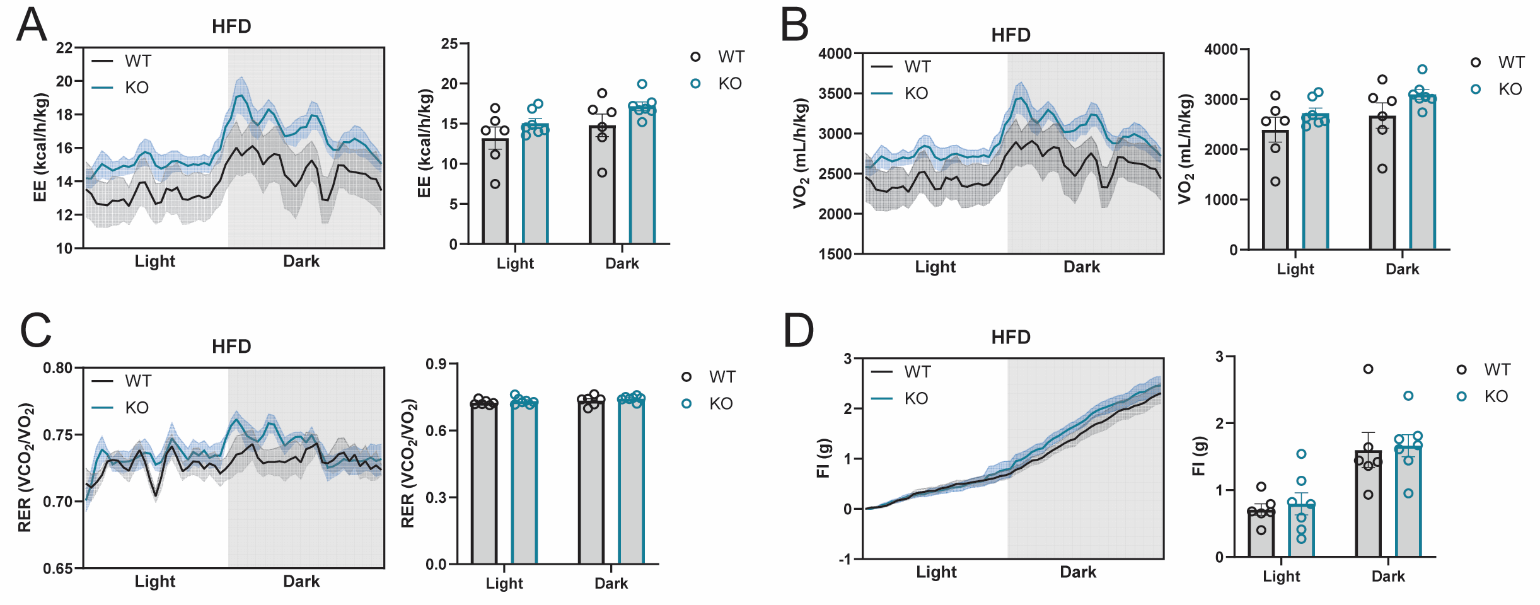


**The metabolic features of Gpr17 germline knockout mice under short-term HFD**

(A-D) Representative mouse energy expenditure (EE), oxygen consumption (VO_2_), respiratory exchange ratio (RER), and food intake (FI) during ad libitum HFD feeding (WT = 6, KO = 7).

.

**Figure S2**


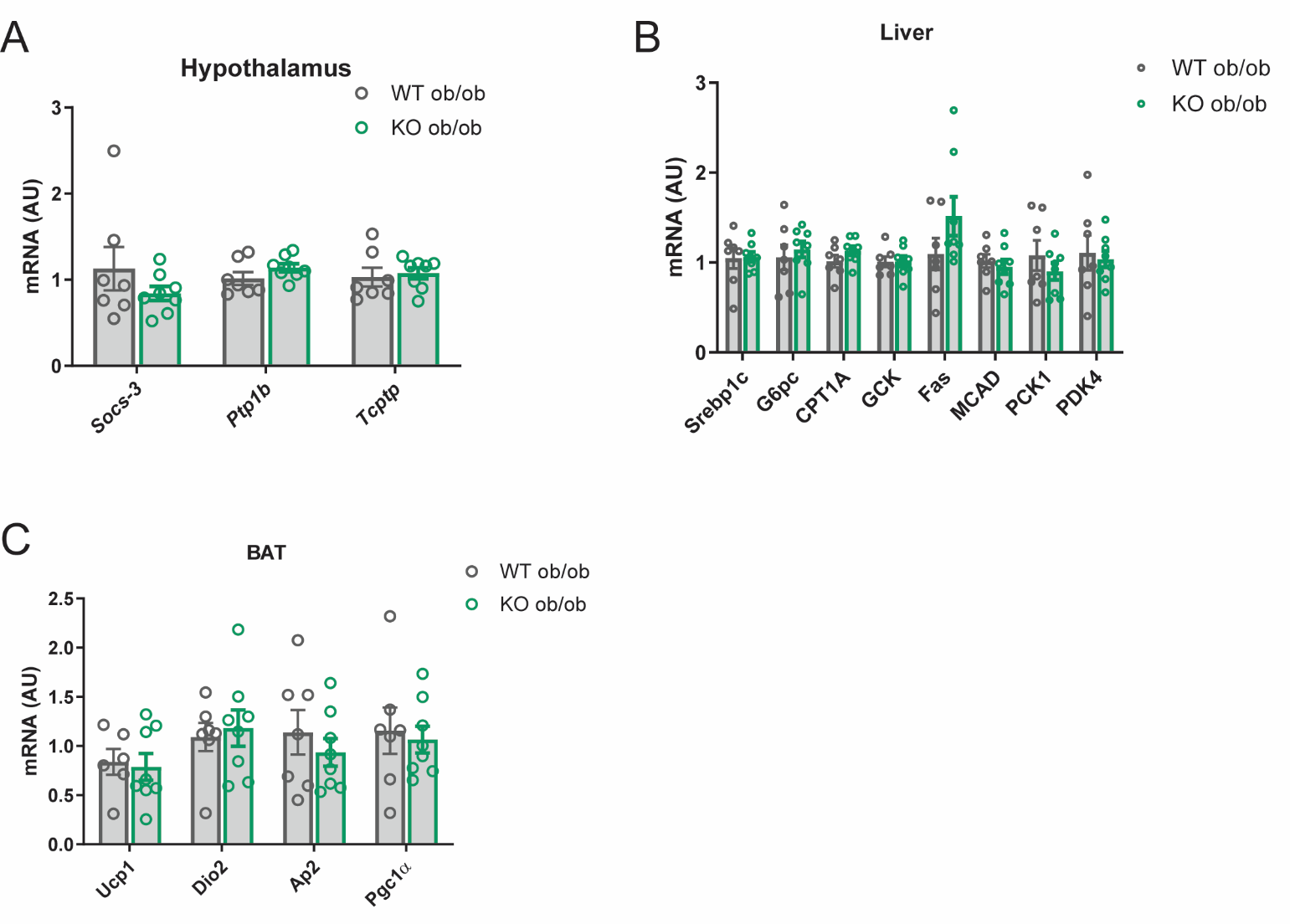


**The mRNA expression in Gpr17 knockout *ob/ob* mice**

(A) The hypothalamic mRNA levels of leptin signaling inhibitors in *ob/ob* Gpr17 knockout mice.

(B) Hepatic mRNA expression of genes involved in glucose and lipid metabolism.

(C) The mRNA levels of thermogenetic genes in brown adipose tissue (BAT).

WT *ob/ob* = 7, KO *ob/ob* = 8. Data were displayed as means ± SEM.

**Figure S3**


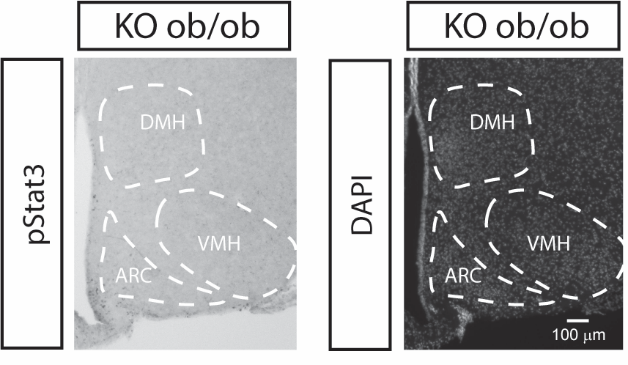


**The representative image of pStat3 immunoreactivity in KO *ob/ob* mice treated with vehicle**
